# Association of neutrophil-to-lymphocyte ratio with all-cause and cardiovascular mortality in patients with circadian rhythm syndrome: A longitudinal cohort study based on NHANES 2005–2018 data

**DOI:** 10.1097/MD.0000000000049416

**Published:** 2026-06-26

**Authors:** Jia Wei, Pengfei Wang, Kun Ma, Ying Chen, Ying Ye

**Affiliations:** a Department of Traditional Chinese Medicine, Fuzhou University Affiliated Provincial Hospital, Fuzhou, Fujian Province, P.R. China.

**Keywords:** all-cause mortality, cardiovascular mortality, circadian rhythm syndromes, neutrophil lymphocyte ratio

## Abstract

Circadian rhythm syndrome (CircS) has been associated with an elevated risk of cardiovascular death. However, there is a scarcity of affordable biomarkers for its assessment. The neutrophil-to-lymphocyte ratio (NLR) is a cost-effective inflammatory marker; however, its long-term prognostic significance in CircS remains to be elucidated. This study examined NLR’s association with all-cause and cardiovascular mortality in CircS patients. Using National Health and Nutrition Examination Survey (2005–2018) data, this analysis leveraged records from 9267 patients with confirmed CircS. Mortality outcomes and underlying causes of death were ascertained by linkage to the National Death Index. Subjects were stratified into elevated and reduced NLR cohorts based on established thresholds. A Cox proportional hazards regression model was employed to assess the relationship between NLR and all-cause and cardiovascular mortality. Additionally, restricted cubic splines were utilized to examine the dose–response relationship between NLR and mortality. Stratified analyses and interaction tests were conducted based on variables such as poverty-to-income ratio, body mass index, age, alcohol consumption, and smoking status. During a median observation period of 83 months, 1494 fatalities occurred among 9267 CircS patients (all-cause mortality: 16.1%), including 427 cardiovascular-specific deaths (cardiovascular mortality: 4.6%). Multivariable Cox proportional hazards modeling revealed that elevated NLR values (>3) were associated with heightened all-cause mortality risk (hazard ratio = 1.69, 95% confidence interval: 1.51–1.89, *P* < .001). The relationship between NLR and all-cause mortality was nonlinear (*P* for nonlinearity < .05), whereas NLR and cardiovascular mortality showed a positive linear correlation (*P* for nonlinearity > .05). Subgroup analyses consistently indicated associations across strata, though the robustness of these findings may be influenced by measurement error in self-reported stratification variables. In this large observational cohort, elevated NLR was independently associated with increased all-cause and cardiovascular mortality in CircS patients after multivariable adjustment. These findings suggest NLR may serve as a candidate prognostic biomarker; however, its clinical utility requires validation in prospective studies due to the inherent limitations of observational design.

## 1. Introduction

Disruption of circadian rhythms is a key factor in metabolic dysregulation, with growing evidence implicating its role in metabolic syndrome (MetS) pathogenesis.^[1]^ Circadian rhythm syndrome (CircS) has been proposed as a distinct high-risk clinical phenotype owing to the broad regulatory influence of circadian rhythms on metabolic, sleep, and mood pathways. The diagnostic criteria for CircS include at least 4 chronic conditions, including hypertension, dyslipidemia, abdominal obesity, and diabetes, along with insufficient sleep and depression.^[2]^ This composite phenotype arises from the pervasive regulatory functions of the circadian rhythms in these pathways. Notably, CircS demonstrates greater predictive value for cardiovascular disease (CVD) than traditional MetS^[3]^ and correlates with increased mortality risk.^[4,5]^ In addition, as core components of CircS, sleep deprivation and depression have been identified as independent risk factors for cardiovascular events and all-cause mortality, a conclusion that has been verified in several large-scale cohort studies,^[5-7]^ thus further confirming that CircS is a unique high-risk phenotype.

Chronic low-grade inflammation represents a key pathological mechanism linking the core components of CircS, metabolic disturbances, sleep disorders, and depression, which directly drives atherosclerosis and CVD progression. Among the various inflammatory markers, the neutrophil-to-lymphocyte ratio (NLR) has emerged as a robust and clinically accessible indicator of systemic inflammation. This ratio integrates 2 fundamental immune responses: neutrophil-driven innate immunity and lymphocyte-mediated adaptive immunity.^[8-10]^ Notably, elevated NLR demonstrates consistent associations with individual CircS components, predicting incident MetS,^[11]^ correlating with sleep disorder severity,^[11]^ and reflecting chronic depression.^[12]^ Of particular concern is the synergistic inflammatory amplification observed in depression-sleep disorder comorbidity.^[13]^ The clinical relevance of NLR was further validated in the Canakinumab Anti-Inflammatory Thrombosis Outcomes Study trial, where it emerged as one of the strongest predictors of cardiovascular events and all-cause mortality.^[11]^ Although studies have explored the relationship between inflammation and mortality in the individual components of CircS, the prognostic value of NLR in patients with CircS has not yet been fully explored.

This study was undertaken to investigate the association between the NLR and both all-cause and cardiovascular mortality among individuals with CircS. We utilized data from the National Health and Nutrition Examination Survey (NHANES) database and the National Death Index covering the period 2005 to 2018. We hypothesized that an elevated NLR is independently associated with an increased risk of all-cause and cardiovascular mortality in patients with CircS. The findings may inform the development of risk stratification strategies and guide targeted interventions for this vulnerable population.

## 2. Materials and methods

### 2.1. Study design and sample

This study analyzed data from the NHANES, a nationally representative survey conducted by the Centers for Disease Control and Prevention to assess the health status of the US population.^[14-20]^ We included data from 7 consecutive cycles (2005–2006 through 2017–2018). This project received institutional approval from the Ethics Review Board of the National Center for Health Statistics (protocols #2005-06, #2011-17, #2018-01), and written informed consent was obtained from all participants. Our analysis used only de-identified data from the public-use dataset, which contains no personal information. From this database, we identified eligible participants with a diagnosis of CircS. Individuals lacking complete survival or essential laboratory data were excluded from the analysis.

### 2.2. Definition of CircS

A CircS diagnosis requires meeting at least 4 out of 7 criteria: a waist circumference of at least 102 cm for men and 88 cm for women, triglycerides of at least 1.7 mmol/L or prescribed lipid-lowering medication, high-density lipoprotein cholesterol below 1.0 mmol/L for men and 1.3 mmol/L for women or prescribed lipid-lowering medication, systolic blood pressure of at least 135 mm Hg, diastolic pressure of at least 85 mm Hg, or prescribed blood pressure medication, fasting blood glucose of at least 100 mg/dL or prescribed blood sugar-lowering medication, <6 hours of sleep, and Depression status was determined using the validated Patient Health Questionnaire-9, which assesses depression symptom presence and severity over a 2-week recall period; scores were dichotomized as no/minimal depression (0–4) versus clinically significant depression (≥5) to define depression status^[21]^ (Table S1, Supplemental Digital Content 1).

### 2.3. Detection and definition of NLR

The NLR was calculated as the ratio of absolute neutrophil count to absolute lymphocyte count, both obtained from standard complete blood count analyses.^[22]^ Using a clinically established NLR cutoff (>3) derived from published literature, participants were stratified into higher-NLR (n = 1661) and lower-NLR (n = 7606) groups. This threshold selection was based on^[23,24]^: Clinical precedence: Established as a validated prognostic marker in cardiometabolic cohorts,^[16,17]^ Biological rationale: Reflects synergistic effects of neutrophilia and lymphocytopenia indicating significant systemic immune dysregulation, and Empirical evidence: The magnitude of mortality risk associated with this cutoff is comparable to that of the highest NLR quartile, with superior clinical utility (Table S2, Supplemental Digital Content 2). Robustness of this approach was further confirmed through continuous NLR analysis and quartile-based stratification in subsequent analyses.

### 2.4. Mortality outcomes of the study population

The determination of mortality status was achieved by linking participant records to the National Death Index between January 1, 2005 and December 31, 2019. This linkage utilized the NHANES Public-Use Linked Mortality Files. All-cause mortality included deaths from any cause, while cardiovascular mortality was identified using International Classification of Diseases, 10th Revision codes I00–I09, I11, I13, and I20–I51, covering various heart-related diseases.

### 2.5. Covariates

Covariates encompassed age groups (<60, ≥60), sex classification (male, female), and racial categories (Mexican American, other Hispanic, non-Hispanic White, non-Hispanic Black, and other). Body mass index (BMI) was segmented as follows: <25 kg/m^2^ (normal), 25–30 kg/m^2^ (overweight), and >30 kg/m^2^ (obese). Educational attainment was divided into 3 levels: below high school, high school, and above high school. Poverty status was assessed using the poverty-to-income ratio (PIR), which was categorized as low (<1.3), middle (1.3–3.5), and high (>3.5). Smoking history was determined by the question, “Have you ever smoked at least 100 cigarettes in your lifetime?” Marital status was categorized as “married” or “unmarried,” with the latter including individuals who reported being never married, widowed, divorced, or separated. Drinking status was classified as <12 drinks/yr, 12 drinks/yr, or >12 drinks/yr. CVD was defined based on a self-reported history of heart disease and/or stroke. Participants were verbally asked if a physician had ever diagnosed them with a heart attack, angina, coronary artery disease, heart failure, or any other heart-related problems for heart disease diagnosis. For stroke diagnosis, they were also asked, “Has a doctor ever told you that you have been diagnosed with a stroke?” Cancer was defined on the basis of self-reported physician-diagnosed results.

### 2.6. Statistical analyses

Continuous variables were presented as mean ± standard deviation, whereas categorical variables were described using percentages. Group comparisons of continuous variables were performed using either the independent samples *t* test or the Mann–Whitney *U* test, selected based on distributional normality assessments. Categorical variables were compared using the chi-square or Fisher exact test, as applicable. We used Cox proportional hazards regression to compute hazard ratios (HR) with 95% confidence intervals (CI), evaluating the association of NLR with all-cause and cardiovascular mortality. To address missing data, a chained-equation approach was employed using multiple imputation with 5 replications. The Kaplan–Meier methodology derived survival probabilities, whereas group wise comparisons relied on log-rank testing. The confounding factors included in the analysis were selected based on clinical expertise and a review of pertinent literature.^[25,26]^ Three different models were included in our multivariate Cox proportional hazards regression model to manage confounding factors. However, the crude analytical approach was not adjusted. Model 1 incorporated age, sex, and race, whereas model 2 further controlled for additional covariates: BMI, education level, PIR, marital status, smoking and drinking behaviors, hypertension, diabetes, cancer history, and CVD.

Restricted cubic spline (RCS) models were employed to construct HR curves, which facilitated the examination of potential nonlinear dose–response relationships between NLR and all-cause mortality. Similar analyses have been conducted to explore their association with cardiovascular mortality.

The effect of NLR on all-cause and cardiovascular mortality was assessed using subgroup and interaction analyses. The analyses considered various stratification factors, including PIR levels (<1.3, 1.3–3.5, >3.5), BMI (<25 kg/m^2^, 25–30 kg/m^2^, ≥30 kg/m^2^), age groups (<60 years, ≥60 years), smoking status (no, yes), and drinking status (no, yes). The significance of the interactions was determined using *P*-values for the interaction terms between NLR and the stratified variables, which were displayed in a forest plot. All analyses were conducted using R (v4.2.2; Beijing Fengrui Kelin Medical Technology Co., Ltd., employing the survival, rms, and forestplot packages for Cox regression, spline fitting, and visualization, respectively) alongside Free Statistics software (v2.2) for data management.

## 3. Results

### 3.1. Characteristics of the study population

**Figure 1.**
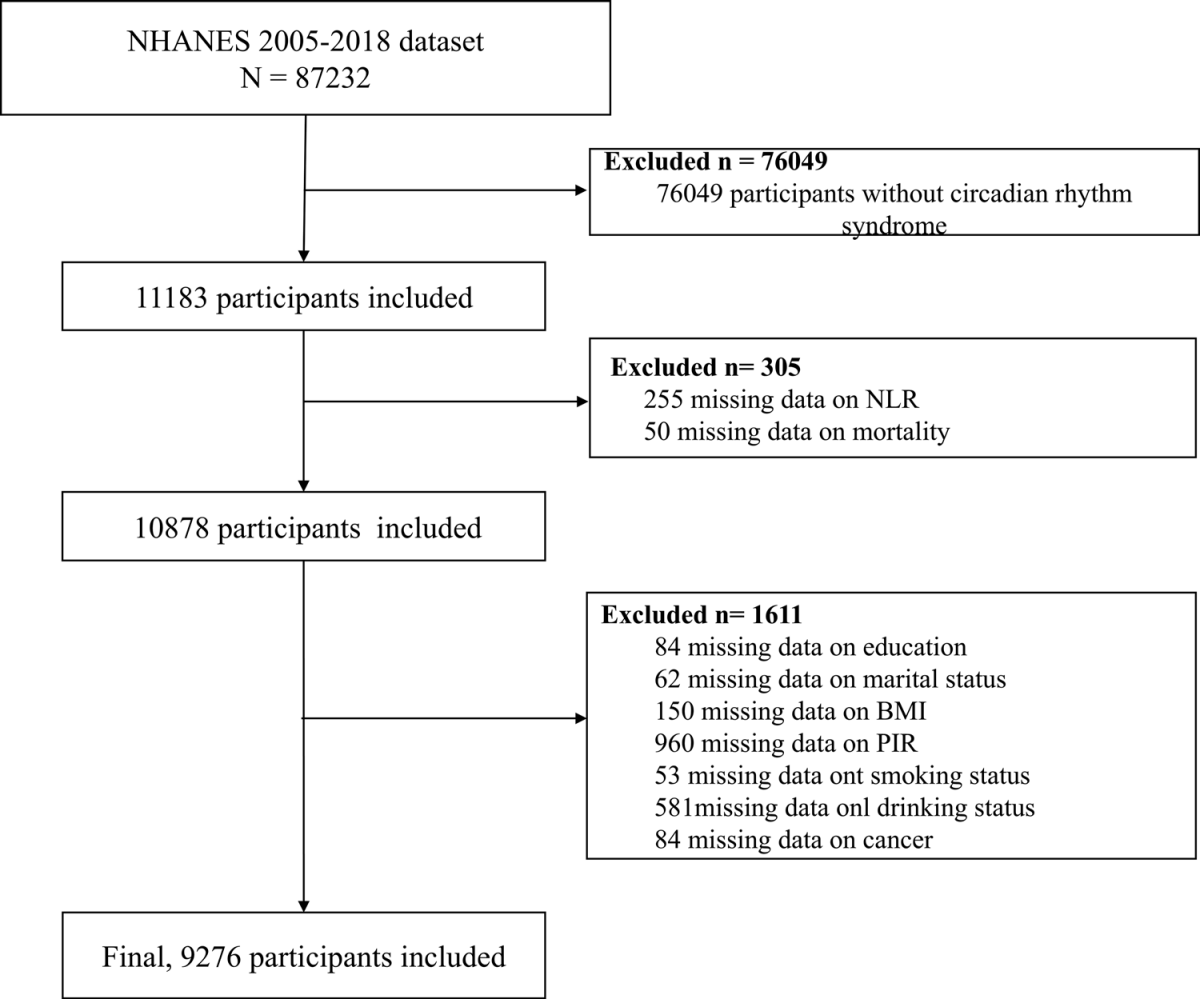
Flow chart of the sample selection from NHANES 2005–2018. BMI = body mass index, NHANES = National Health and Nutrition Examination Survey, NLR = neutrophil-to-lymphocyte ratio, PIR = poverty-to-income ratio.

The final cohort comprised 9267 participants with complete data (Fig. 1). Applying an NLR cutoff of 3, participants were categorized into a higher NLR group (n = 1661) and a lower NLR group (n = 7606; Table 1). During a median observation period of 83 months, 1494 fatalities occurred among 9267 patients (all-cause mortality: 16.1%), including 427 cardiovascular-specific deaths (cardiovascular mortality: 4.6%). Compared to the lower NLR group, the higher NLR group was significantly different in terms of demographic and behavioral characteristics: they were older, predominantly male, more often non-Hispanic White, married, and had higher PIR. Furthermore, self-reported data indicated higher frequencies of smoking and alcohol consumption, as well as higher education levels in this group. However, behavioral variables based on self-report may be subject to recall bias and social desirability bias. We excluded 78,965 participants based on the following criteria: undiagnosed CircS status (n = 76,049, thus encompassing all individuals without CircS), unavailable NLR measurements (n = 255), missing mortality documentation (n = 50), and incomplete covariate data (n = 1611). The proportion of missing data for each covariate is detailed in Table S3, Supplemental Digital Content 3. This exclusion therefore covered all non-CircS individuals and participants with missing data for NLR, mortality, or key covariates.

**Table 1:** Baseline characteristics of participants.

Variables	Total (n = 9267)	Lower NLR (n = 7606)	Higher NLR (n = 1661)	*P*-value
Sex, n (%)				<.001
Male	4310 (46.5)	3406 (44.8)	904 (54.4)	
Female	4957 (53.5)	4200 (55.2)	757 (45.6)	
Age (yr)	58.8 ± 14.9	58.1 ± 14.7	62.5 ± 15.2	<.001
<60	4234 (45.7)	3634 (47.8)	600 (36.1)	
≥60	5033 (54.3)	3972 (52.2)	1061 (63.9)	
Education level, n (%)				.641
Less than high school	2659 (28.7)	2188 (28.8)	471 (28.4)	
High school	2337 (25.2)	1903 (25.0)	434 (26.1)	
Above high school	4271 (46.1)	3515 (46.2)	756 (45.5)	
Marital status, n (%)				.212
No	5399 (58.3)	4454 (58.6)	945 (56.9)	
Yes	3868 (41.7)	3152 (41.4)	716 (43.1)	
Race, n (%)				<.001
Mexican American	1352 (14.6)	1166 (15.3)	186 (11.2)	
Non-Hispanic White	4289 (46.3)	3280 (43.1)	1009 (60.7)	
Non-Hispanic Black	2075 (22.4)	1845 (24.3)	230 (13.8)	
Other	1551 (16.7)	1315 (17.3)	236 (14.2)	
PIR, n (%)	2.4 ± 1.6	2.4 ± 1.6	2.3 ± 1.5	.116
Low	3164 (34.1)	2597 (34.1)	567 (34.1)	.097
Median	3616 (39.0)	2936 (38.6)	680 (40.9)	
High	2487 (26.8)	2073 (27.3)	414 (24.9)	
BMI, n (%)	32.5 ± 7.1	32.5 ± 6.9	32.4 ± 7.9	.866
<25	940 (10.1)	750 (9.9)	190 (11.4)	.023
≥25–30	2822 (30.5)	2291 (30.1)	531 (32.0)	
>30	5505 (59.4)	4565 (60.0)	940 (56.6)	
Drinking status, n (%)				.071
No	2702 (29.2)	2248 (29.6)	454 (27.3)	
Yes	6565 (70.8)	5358 (70.4)	1207 (72.7)	
Cancer, n (%)				<.001
No	7933 (85.6)	6634 (87.2)	1299 (78.2)	
Yes	1334 (14.4)	972 (12.8)	362 (21.8)	
Smoking status, n (%)				<.001
No	4413 (47.6)	3745 (49.2)	668 (40.2)	
Yes	4854 (52.4)	3861 (50.8)	993 (59.8)	
CVD, n (%)				<.001
No	7204 (77.7)	6098 (80.2)	1106 (66.6)	
Yes	2063 (22.3)	1508 (19.8)	555 (33.4)	

BMI = body mass index, CVD = cardiovascular disease, NLR = neutrophil-to-lymphocyte ratio, PIR = poverty-to-income ratio.

### 3.2. Associations of the NLR with all‑cause mortality

**Figure 2.**
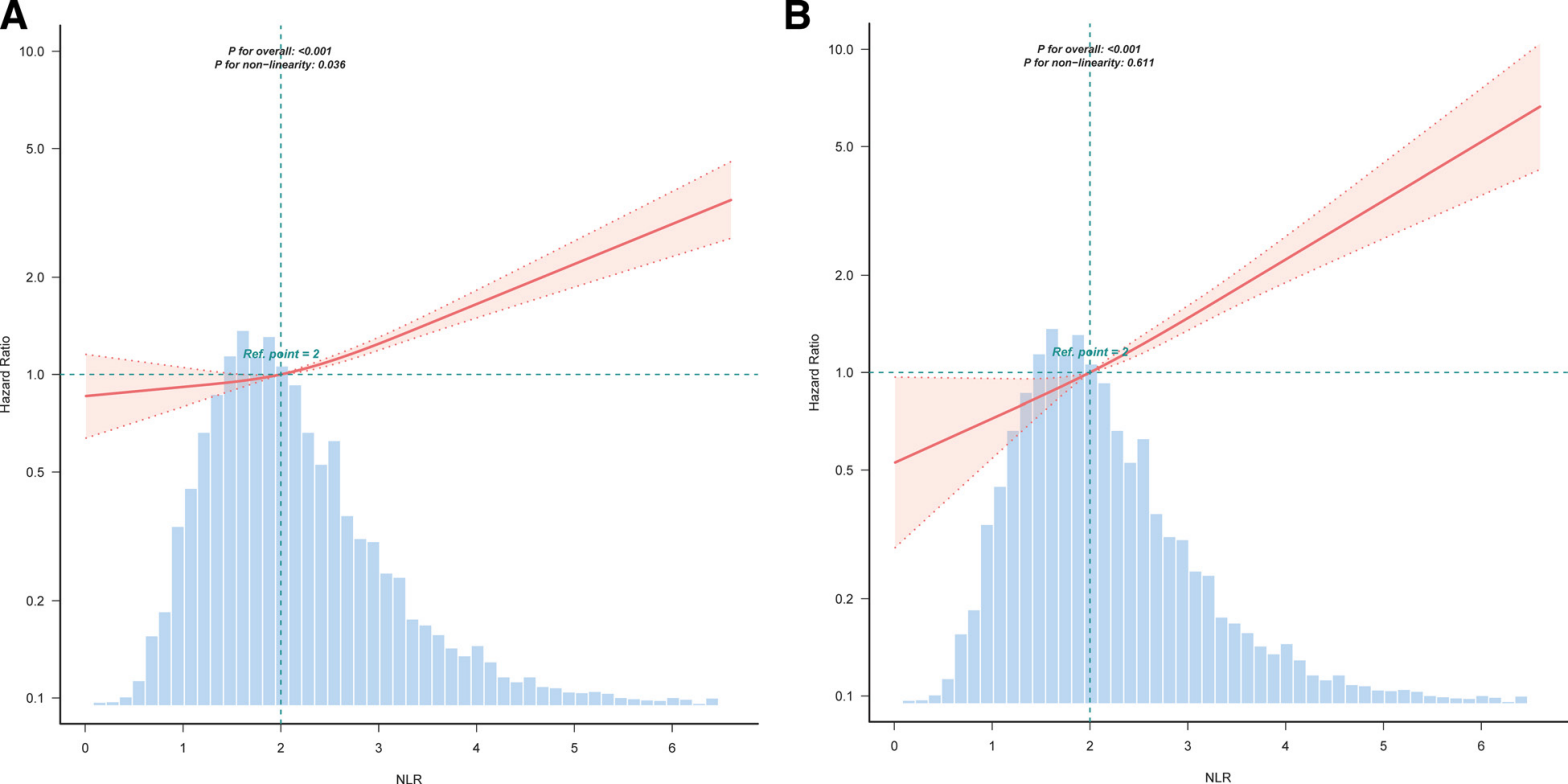
The association between NLR and all-cause (A) and cardiovascular mortality (B) in patients with CircS visualized using restricted cubic splines. Hazard ratios were adjusted for age, sex, race, BMI, education level, PIR, marital status, smoking status, drinking status, cancer, and CVD. The *P*-value for nonlinearity of all-cause mortality was .036. BMI = body mass index, CVD = cardiovascular disease, NLR = neutrophil-to-lymphocyte ratio, PIR = poverty-to-income ratio.

During a median 83-month follow-up of the cohort (n = 9267), 1494 all-cause deaths (16.1%) occurred. Analysis revealed a nonlinear dose–response relationship between NLR and all-cause mortality in patients with CircS (*P* nonlinearity = .036; Fig. 2A). In multivariable Cox regression analyses, each unit increase in NLR was associated with a 16% higher mortality risk in unadjusted analysis (HR: 1.16, 95% CI: 1.14–1.17, *P* < .001), which remained elevated by 13% following multivariable adjustment (model 2 – HR: 1.13, 95% CI: 1.11–1.15, *P* < .001; Table 2). Given that covariates including self-reported diagnoses and behaviors may introduce misclassification bias, the adjusted estimates should be interpreted with caution regarding residual confounding. For categorical NLR analysis, the higher-NLR group exhibited a 2.35-fold increase in crude mortality risk (95% CI: 2.10–2.62), which persisted as a 1.69-fold higher risk after adjustment (HR 1.69, 95% CI: 1.51–1.89; Table 2). The correlation between NLR and all-cause mortality persisted following multiple imputation for missing covariates (Table S4, Supplemental Digital Content 4).

**Table 2:** The correlation between neutrophil-to-lymphocyte ratio and mortality in circadian rhythm syndrome.

Characteristic	Crude model*		Model 1†		Model 2‡	
	HR (95% CI)	*P*-value	HR (95% CI)	*P*-value	HR (95% CI)	*P*-value
All-cause mortality						
NLR	1.16 (1.14–1.17)	<.001	1.14 (1.12–1.16)	<.001	1.13 (1.11–1.15)	<.001
NLR category						
Lower NLR (n = 7606)	1 (Ref)		1 (Ref)		1 (Ref)	
Higher NLR (n = 1661)	2.35 (2.10–2.62)	<.001	1.93 (1.72–2.16)	<.001	1.69 (1.51–1.89)	<.001
Cardiovascular mortality						
NLR	1.18 (1.15–1.20)	<.001	1.16 (1.13–1.19)	<.001	1.14 (1.11–1.17)	<.001
NLR category						
Lower NLR (n = 7606)	1 (Ref)		1 (Ref)		1 (Ref)	
Higher NLR (n = 1661)	3.21 (2.64–3.90)	<.001	2.62 (2.14–3.20)	<.001	2.21 (1.81–2.70)	<.001

BMI = body mass index, CI = Confidence interval, CVD = cardiovascular disease, HR = hazard ratio, NLR = neutrophil-to-lymphocyte ratio, PIR = poverty-to-income ratio.

* Crude model: no other covariates were adjusted.

† Model 1: age, sex, and race.

‡ Model 2: age, sex, race, BMI, education level, PIR, marital status, smoking status, drinking status, cancer, and CVD.

**Figure 3.**
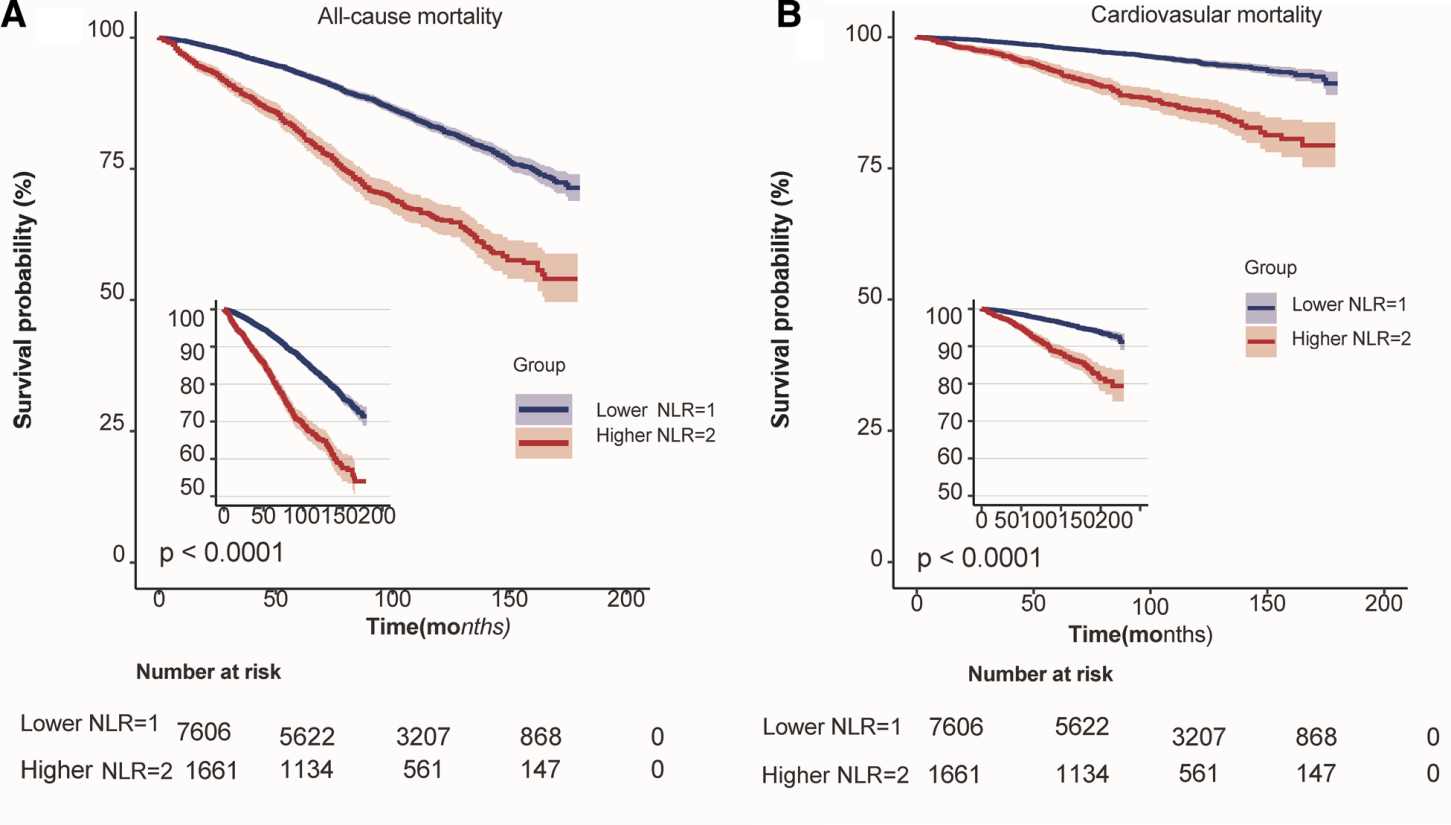
Kaplan–Meier curves for all-cause mortality (A) and cardiovascular mortality (B) by neutrophil-to-lymphocyte ratio level in adults with circadian rhythm syndrome. NLR = neutrophil-to-lymphocyte ratio.

**Figure 4.**
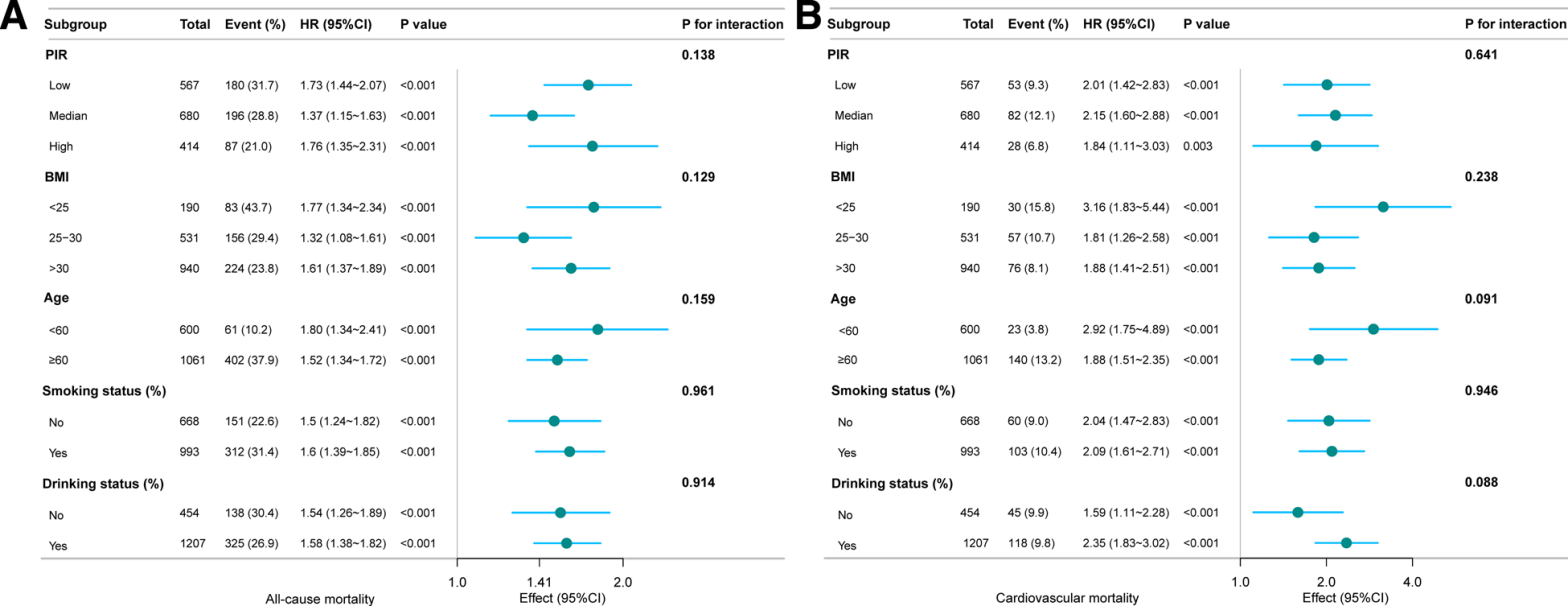
Forest plot showing the association of neutrophil-to-lymphocyte ratio with all-cause (A) and cardiovascular mortality (B) in patients with circadian rhythm syndrome (NHANES 2005–2018). Hazard ratios and 95 % confidence intervals are presented for the overall cohort and various subgroups. All models were adjusted for age, sex, race, BMI, education level, PIR, marital status, smoking status, drinking status, cancer, and CVD, except when the variable itself was the stratification factor. BMI = body mass index, CI = confidence interval, CVD = cardiovascular disease, HR = hazard ratio, NHANES = National Health and Nutrition Examination Survey, PIR = poverty-to-income ratio.

Kaplan–Meier analysis demonstrated significantly divergent survival curves between higher and lower NLR groups (*P* < .001), with markedly reduced survival in the higher NLR cohort (Fig. 3A). Segmented regression identified a clinically significant NLR threshold at 2.608. Above this threshold, each unit NLR increase was associated with 34% higher all-cause mortality risk (HR: 1.34, 95% CI: 1.25–1.44; *P* < .001; Table 3), whereas below 2.608, the association was nonsignificant (HR: 1.14, 95% CI: 1.00–1.31; *P* = .055; Table 3). This threshold effect likely reflects accelerated inflammatory damage accumulation. Subgroup analyses stratified by age, PIR, BMI, smoking status, and alcohol consumption consistently demonstrated this association across all populations (Fig. 4A), with no significant interaction effects observed (all *P*-interaction >.05). The null interaction effects should be interpreted considering potential non-differential misclassification in self-reported stratifying variables, which may attenuate true interaction magnitudes.

**Table 3:** Threshold effect analysis of the neutrophil-to-lymphocyte ratio on all-cause mortality in patients with circadian rhythm syndrome.

Threshold of NLR	HR	95% CI	*P*-value
<2.608	1.14	1.00–1.31	.055
≥2.608	1.34	1.25–1.44	<.001

Adjusted for: age, sex, race, BMI, education level, PIR, marital status, smoking status, drinking status, cancer, and CVD.

BMI = body mass index, CI = confidence interval, CVD = cardiovascular disease, HR = hazard ratio, NLR = neutrophil-to-lymphocyte ratio, PIR = poverty-to-income ratio.

### 3.3. Associations of the NLR with cardiovascular mortality

The analytical cohort comprised 9267 participants for assessing NLR associations with cardiovascular mortality (n = 427). RCS analysis indicated a positive linear relationship between NLR and cardiovascular mortality in CircS patients (*P* nonlinearity =.694; Fig. 2B). In multivariable Cox regression analyses, each NLR unit increment conferred 18% higher cardiovascular mortality risk (HR: 1.18, 95% CI: 1.15–1.20, *P* < .001). After full multivariable adjustment (model 2), this association attenuated but remained significant (model 2 – HR: 1.14, 95% CI: 1.11–1.17, *P* < .001; Table 2). Similarly, the high-NLR group exhibited a 3.21-fold higher crude cardiovascular mortality risk (crude model – 95% CI: 2.64–3.90) which decreased to 2.21-fold after adjustment (model 2 – 95% CI: 1.81–2.70; *P* < .001; Table 2). The association between NLR and cardiovascular mortality maintained its stability following multiple imputation for incomplete data (Table S4, Supplemental Digital Content 4) Survival curves diverged significantly between higher and lower NLR groups (*P* < .001), showing reduced cardiovascular survival probability in the higher-NLR cohort (Fig. 3B). The association remained consistent across all subgroups (Fig. 4B) without significant interaction effects (all *P*-interaction >.05).

## 4. Discussion

This large-scale cohort analysis suggests robust evidence that the NLR functions as an independent factor associated with all-cause and cardiovascular mortality in individuals with CircS. A significant positive association between NLR and both endpoints persisted following extensive adjustment for multiple covariates, though residual confounding due to covariate measurement limitations (e.g., self-reported data) cannot be excluded. RCS modeling revealed a nonlinear relationship for all-cause mortality, with an inflection point at 2.608. Notably, this association remained consistent across the key subgroups stratified by PIR, BMI, age, alcohol consumption, and smoking status. These findings imply that NLR may serve as an epidemiological indicator of risk in CircS populations; however, its consideration as a clinical prognostic biomarker requires cautious interpretation and further validation, especially given reliance on self-reported data.

The biological plausibility of this association is supported by the role of neutrophils and lymphocytes in systemic inflammation. Neutrophils, which are primary responders in host immunity, significantly affect chronic inflammatory diseases,^[27]^ and their counts rise during systemic inflammation due to inhibited apoptosis.^[28,29]^ Conversely, lymphocyte apoptosis increases under physical stress^[30,31]^ or conditions such as hyperglycemia and diabetes mellitus (DM).^[32]^ The NLR, which integrates these opposing trends, offers a potentially more accurate gauge of systemic inflammation severity than individual leukocyte counts and is less susceptible to confounding.^[33,34]^ This is particularly relevant for CircS, which is linked to immune activation and persistent inflammation.^[22]^ Elevated NLR in diabetes also reflects the inflammatory burden,^[22,35,36]^ and NLR is increasingly recognized as a risk factor for cardiovascular mortality, depression, and sleep disorders. Our findings extend this evidence by demonstrating the prognostic association of NLR with mortality risk in CircS, though causality cannot be inferred from this observational design.

Circadian disruption promotes chronic inflammation via the HPA axis and autonomic imbalance, contributing to elevated mortality risk. However, practical biomarkers for quantifying inflammation and predicting mortality in CircS are lacking. While prior studies link NLR to circadian-related conditions such as depression (positively correlated with HAM-D scores^[12]^), sleep disorders (acting as an independent risk factor and mediator linking sleep disorders to depression^[13]^), and MetS (positively correlated with severity, with NLR ≥ 1.67 proposed as a US screening cutoff^[11]^), no study has established NLR’s independent prognostic association with mortality in CircS. Our analysis addresses this gap but requires replication in prospective cohorts. Using a cohort of 9267 patients diagnosed with CircS, we demonstrated that elevated NLR remained independently associated with increased all-cause and cardiovascular mortality risk, even after extensive multivariable adjustment. The underlying mechanism likely involves neutrophilia exacerbating chronic inflammation coupled with lymphopenia, impairing immune defenses, and collectively diminishing host immunity and resistance.^[37]^

Specifically, in this population-based study, the NLR exhibited a nonlinear relationship with all-cause mortality and a positive linear relationship with cardiovascular mortality. Multivariable Cox regression (fully adjusted) showed that individuals in the higher NLR group faced a 69% higher risk of all-cause death and a 121% higher risk of cardiovascular death than those in the lower NLR group. A nonlinear association emerged between NLR and all-cause mortality risk, marked by a distinct threshold effect at NLR = 2.608. Above this inflection point, mortality risk accelerated significantly (*P *< .01). This inflection value may reflect cumulative subclinical cardiovascular injury from chronic inflammation. While NLR > 2.608 emerged as a threshold for accelerated risk in our statistical model, we caution against the direct clinical application of this cutoff. Its utility as a clinical risk indicator requires rigorous external validation, especially in cohorts with objectively measured covariates rather than self-reported data.

The primary merits of this study are its substantial sample size and extended follow-up duration, which collectively contribute to the robustness of the conclusions and effectively minimize selection bias among the NHANES participants. Although multiple covariates were considered and adjusted for in our multivariable model, residual confounding may remain due to unmeasured or insufficiently adjusted variables. Notably, data on chronic or acute infections, specific inflammatory disorders, detailed medication use (e.g., anti-inflammatory or immunomodulatory drugs), and objective physical activity measures were either lacking or incomplete in the NHANES dataset. These unmeasured confounders could independently affect both NLR and mortality risk, compromising causal inference. Consequently, results should be interpreted cautiously. Moreover, self-reported physician diagnosis information included in covariates may be subject to recall bias and misclassification bias, with its limitations requiring careful consideration during clinical interpretation. It should be emphasized that while these limitations do not compromise the internal validity of our study, the generalizability of findings – derived primarily from a US cardiovascular disease cohort – requires validation across diverse populations. Our results suggest NLR may serve as a potential independent prognostic indicator for mortality risk in CVDs, yet its clinical utility warrants verification in studies incorporating more refined phenotypic data. Future research should evaluate the clinical value of NLR-based risk stratification strategies and compare the predictive efficacy of serial NLR measurements versus single baseline assessments.

## 5. Conclusion

In this large cohort of patients with CircS, elevated NLR was independently associated with higher risks of all-cause and cardiovascular mortality after adjustment for key covariates. NLR may represent a promising mechanistic target to mitigate premature mortality in this population. However, limitations related to self-reported data and residual confounding necessitate cautious interpretation of its prognostic value. Further validation via well-designed prospective studies focused on causal inference and intervention effects is warranted.

## Acknowledgments

The authors gratefully acknowledge the NHANES participants and research personnel for their invaluable contributions to data collection and study implementation.

## Author contributions

**Conceptualization:** Kun Ma, Ying Chen.

**Formal analysis:** Jia Wei.

**Methodology:** Pengfei Wang.

**Software:** Ying Ye.

**Writing – original draft:** Jia Wei, Ying Chen.

**Writing – review & editing:** Jia Wei, Pengfei Wang, Kun Ma, Ying Ye.








