# Supplementary material for: Association of neutrophil-to-lymphocyte ratio with all-cause and cardiovascular mortality in patients with circadian rhythm syndrome: A longitudinal cohort study based on NHANES 2005–2018 data
Source: Medicine (Baltimore). 2026 Jun 26;105(26):e49416. doi: 10.1097/MD.0000000000049416 (PMC13313709; doi:10.1097/MD.0000000000049416)
Supplement: Supplementary file 1 [file medi-105-e49416-s001.docx]

**Supplementary Table S1.** Detailed descriptions and scoring criteria of each PHQ-9 item in NHANES 2005-2018 questionnaire.

| **Characteristics** | **Descriptions** | **Answers and scoring criteria** |
| --- | --- | --- |
| PHQ-9 item 1 | Little interest in doing things | Not at all - score 0  Several days - score 1  More than half the days - score 2  Nearly every day - score 3 |
| PHQ-9 item 2 | Feeling down, depressed, or hopeless |  |
| PHQ-9 item 3 | Trouble sleeping or sleeping too much |  |
| PHQ-9 item 4 | Feeling tired or having little energy |  |
| PHQ-9 item 5 | Poor appetite or overeating |  |
| PHQ-9 item 6 | Feeling bad about yourself |  |
| PHQ-9 item 7 | Trouble concentrating on things |  |
| PHQ-9 item 8 | Moving or speaking slowly or too fast |  |
| PHQ-9 item 9 | Thought you would be better off dead |  |

NHANES = National Health and Nutrition Examination Survey; PHQ-9 = Patient Health Questionnaire-9.
