# Supplementary material for: Association of neutrophil-to-lymphocyte ratio with all-cause and cardiovascular mortality in patients with circadian rhythm syndrome: A longitudinal cohort study based on NHANES 2005–2018 data
Source: Medicine (Baltimore). 2026 Jun 26;105(26):e49416. doi: 10.1097/MD.0000000000049416 (PMC13313709; doi:10.1097/MD.0000000000049416)
Supplement: Supplementary file 2 [file medi-105-e49416-s002.docx]

**Supplementary Table S2.** The Correlation Between Neutrophil-to-Lymphocyte Ratio and Mortality in Circadian Rhythm Syndrome.

| **Quartiles** |  | **Crude Model^a^** | | **Model1^b^** | | **Model2^c^** | |
| --- | --- | --- | --- | --- | --- | --- | --- |
|  | **No** | **HR (95%CI)** | ***P* value** | **HR (95%CI)** | ***P* value** | **HR (95%CI)** | ***P* value** |
| **All-cause mortality** | | |  |  |  |  |  |
| NLR (quartile) |  |  |  |  |  |  |  |
| Q1 | 2288 | 1(Ref) |  | 1(Ref) |  | 1(Ref) |  |
| Q2 | 2151 | 1.13 (0.95~1.34) | 0.165 | 1.1 (0.92~1.30) | 0.296 | 1.07 (0.90~1.28) | 0.420 |
| Q3 | 2507 | 1.35 (1.15~1.58) | < 0.001 | 1.21 (1.03~1.43) | 0.020 | 1.16 (0.99~1.37) | 0.068 |
| Q4 | 2321 | 2.48 (2.14~2.87) | < 0.001 | 1.97 (1.69~2.30) | < 0.001 | 1.75 (1.50~2.04) | < 0.001 |
| *P* for trend | 9627 |  | < 0.001 |  | < 0.001 |  | < 0.001 |
| **Cardiovascular mortality** | | |  |  |  |  |  |
| NLR (quartile) |  |  |  |  |  |  |  |
| Q1 | 2288 | 1 (Ref) |  | 1 (Ref) |  | 1 (Ref) |  |
| Q2 | 2151 | 1.19 (0.83~1.70) | 0.334 | 1.18 (0.82~1.69) | 0.376 | 1.14 (0.79~1.63) | 0.489 |
| Q3 | 2507 | 1.67 (1.21~2.30) | 0.002 | 1.52 (1.09~2.11) | 0.013 | 1.42 (1.02~1.98) | 0.037 |
| Q4 | 2321 | 3.81 (2.84~5.12) | < 0.001 | 3.07 (2.26~4.16) | < 0.001 | 2.61 (1.92~3.55) | < 0.001 |
| *P* for trend | 9627 |  | < 0.001 |  | < 0.001 |  | < 0.001 |

**^a^**Crude Model: no other covariates were adjusted;

**^b^**Model 1: age, sex, and race;

**^c^**Model 2: age, sex, race, BMI, education level, PIR, marital status, smoking status, drinking status, cancer, and CVD.

HR = hazard ratio; CI = confidence interval; BMI = body mass index; CVD = cardiovascular disease; PIR = poverty-to-income ratio; NLR = neutrophil-to-lymphocyte ratio.
