# Supplementary material for: Association of neutrophil-to-lymphocyte ratio with all-cause and cardiovascular mortality in patients with circadian rhythm syndrome: A longitudinal cohort study based on NHANES 2005–2018 data
Source: Medicine (Baltimore). 2026 Jun 26;105(26):e49416. doi: 10.1097/MD.0000000000049416 (PMC13313709; doi:10.1097/MD.0000000000049416)
Supplement: Supplementary file 3 [file medi-105-e49416-s003.docx]

**Supplementary Table S3.** Baseline Variable Missing-Data Summary, NHANES 2005–2018 **(n = 10 878)**

| **Variable** | **Number of Missing** | **Missing Rate, n (%)** |
| --- | --- | --- |
| Age | 0 | 0.0 |
| Sex | 0 | 0.0 |
| Race | 0 | 0.0 |
| BMI | 150 | 1.38% |
| CVD | 0 | 0.00 |
| Cancer | 84 | 0.77% |
| Education level | 84 | 0.77% |
| Marital status | 54 | 0.50% |
| Smoking status | 53 | 0.49% |
| Drinking status | 581 | 5.34% |
| PIR | 960 | 8.83% |

BMI = body mass index; CVD = cardiovascular disease; PIR = poverty-to-income ratio.
