# Supplementary material for: Association of neutrophil-to-lymphocyte ratio with all-cause and cardiovascular mortality in patients with circadian rhythm syndrome: A longitudinal cohort study based on NHANES 2005–2018 data
Source: Medicine (Baltimore). 2026 Jun 26;105(26):e49416. doi: 10.1097/MD.0000000000049416 (PMC13313709; doi:10.1097/MD.0000000000049416)
Supplement: Supplementary file 4 [file medi-105-e49416-s004.docx]

**Supplementary Table S4.** The Correlation Between Neutrophil-to-Lymphocyte Ratio and Mortality in Circadian Rhythm Syndrome after multiple imputation.

| **Characteristic** | **Crude Model^a^** | | **Model1^b^** | | **Model2^c^** | |
| --- | --- | --- | --- | --- | --- | --- |
|  | **HR (95%CI)** | ***P* value** | **HR (95%CI)** | ***P* value** | **HR (95%CI)** | ***P* value** |
| **All-cause mortality** |  |  |  |  |  |  |
| NLR(n=10878) | 1.17 (1.15~1.18) | < 0.001 | 1.14 (1.13~1.16) | < 0.001 | 1.13 (1.11~1.15) | < 0.001 |
| **NLR category** |  |  |  |  |  |  |
| Lower NLR (n = 8918) | 1 (Ref) |  | 1 (Ref) |  | 1 (Ref) |  |
| Higher NLR (n = 1960) | 2.44 (2.21~2.7) | < 0.001 | 2 (1.81~2.22) | < 0.001 | 1.73 (1.56~1.92) | < 0.001 |
| **Cardiovascular mortality** |  |  |  |  |  |  |
| NLR(n=10878) | 1.18 (1.16~1.21) | < 0.001 | 1.16 (1.13~1.19) | < 0.001 | 1.14 (1.11~1.17) | < 0.001 |
| **NLR category** |  |  |  |  |  |  |
| Lower NLR (n = 8918) | 1 (Ref) |  | 1 (Ref) |  | 1 (Ref) |  |
| Higher NLR (n = 1960) | 3.29 (2.74~3.95) | < 0.001 | 2.69 (2.23~3.24) | < 0.001 | 2.24 (1.85~2.70) | < 0.001 |

**^a^**Crude Model: no other covariates were adjusted;

**^b^**Model 1: age, sex, and race;

**^c^**Model 2: age, sex, race, BMI, education level, PIR, marital status, smoking status, drinking status, cancer, and CVD.

HR = hazard ratio; CI = confidence interval; BMI = body mass index; CVD = cardiovascular disease; PIR = poverty-to-income ratio; NLR = neutrophil-to-lymphocyte ratio.
